# Remarks on Leaden Pipes

**Published:** 1853-11

**Authors:** 


*Remarks on Leaden Pipes. With an Account of a Cheap Method of rendering Leaden Pipes harmless.*—We give place to the following letter, although dissenting from so much of it as ascribes disease to the lead detected in the Croton water when passing through leaden pipes. It must stand in leaden vessels long enough for mutual decomposition, before any deterioration of the water can take place, at the mean temperature, as every chemist knows. As to the workers in lead, who inhale it in vapors from the salts of the metal, and their diseases, every body understands that subject. And as to the weak brethren with female nerves, who "drink pounded ice at the Carlton, for fear of the lead in Croton water," the protective plan suggested by our correspondent may serve their turn. We publish it, that both sides may have a hearing:

At the time of the introduction of the Croton water, a strong effort was made to direct public attention to the employment of tinned leaden pipes as a means for its conveyance, but the interests of plumbers and contractors were too great to allow the general use of other than the cheapest materials.

1. It is admitted that lead is a poison of a slow and insidious nature, producing diseases the cause of which is often unknown both to the physician and patient. The effects are very apparent when individuals have been steadily exposed to its influence as plumbers, painters, &c.

2. Although taken in small quantities, it has the power of accumulating in the system, and of ultimately causing serious complaints, such as habitual constipation, palsy, blindness, deafness, colic, &c., just as a few drops of the tincture of foxglove, when repeated as often as we take our meals, produces in some constitutions loss of appetite, want of sleep and diminution of the pulse. A solution of arsenic, without either taste or smell, may, under the same circumstances, occasion tenderness of the skin, dropping off of the nails, and according to the distinguished Broussais, lay the foundation of some chronic disease. Minute quantities of iodine taken daily will sometimes cause falling out of the hair, shriveling up of certain organs and deprivation of their functions. The cumulative power of mercury has been long established. Calomel in a dose of twenty grains acts as a purgative, whereas less than a fourth of that quantity insidiously introduced by divided doses, may occasion salivation. In like manner, the severe colic, the paralytic affections, &c., of artisans in lead are not usually produced at once; the poison enters the system by slow degrees, and the individual is unconscious of danger until enough has been taken to produce the effects.

There is in the constitution a sanative influence or effort to throw off that which is noxious, and this is greater in some constitutions than in others.

We may meet with healthy drunkards at sixty or seventy, but in most of these that age is not reached; there are some who cannot be affected with mercury, yet there are others whom a single grain will salivate. If individuals of a healthy constitution resist the effects of lead, all are not alike exempt.

3. The observations of Lambe, and the experiments of Christison, show that lead is more rapidly corroded by pure than impure water. If it would be permitted to introduce into the reservoir a quantity of chloride of iodide of sodium, or an alkaline phosphate, such water would not be deteriorated by passing through lead.

4. It is astonishing with what perversity plumbers and others will affirm that lead is perfectly harmless; that coating it with tin is a failure; that tinned leaden pipe has been dug up full of holes; and that tin by being negatively electric with respect to lead, causes the latter metal to dissolve more rapidly. That lead is not harmless is an established fact. If an imperfectly tinned tube has been found full of holes, that proves nothing, for in the Leverian Museum there is a piece of lead with holes in it formed by rain water. Silver is negative with respect to copper, but copper completely covered with silver is not so likely to corrode as when it is uncovered. Gold is among the most negative of the metals, yet it is used without corroding the material which it covers. Tin is negative to iron, but the latter metal is not corroded on that account, as we may observe by the extensive use of tinned plates.

*Cheap method of rendering Leaden Pipes harmless.*—As already stated, when an alkaline chloride is introduced into a leaden pipe, the inside of the tube becomes coated with the chloride of lead, which is insoluble in water. By turning off the water from the main pipe and soldering on a stop-cock, to which a small pump is attached, a solution of common salt may be pumped from the uppermost story into every tube in the house. In warm weather, a day, or two days at most, would be sufficient to give the pipes an insoluble covering. W. C. W.

—*New York Medical Gazette.*

